# Gene–microRNA Network Analysis Identified Seven Hub Genes in Association with Progression and Prognosis in Non-Small Cell Lung Cancer

**DOI:** 10.3390/genes13081480

**Published:** 2022-08-19

**Authors:** Zhiyuan Yang, Hongqi Wang, Zixin Zhao, Yunlong Jin, Zhengnan Zhang, Jiayi Tan, Fuyan Hu

**Affiliations:** 1 School of Artificial Intelligence, Hangzhou Dianzi University, Hangzhou 310018, China; 2 Department of Statistics, Faculty of Science, Wuhan University of Technology, 122 Luoshi Road, Wuhan 430070, China

**Keywords:** non-small cell lung cancer, biomarker, microRNA–gene interaction network

## Abstract

Introduction: Lung cancer is the leading cause of cancer deaths in the world and is usually divided into non-small cell lung cancer (NSCLC) and small cell lung cancer. NSCLC is dominant and accounts for 85% of the total cases. Currently, the therapeutic method of NSCLC is not so satisfactory, and thus identification of new biomarkers is critical for new clinical therapy for this disease. Methods: Datasets of miRNA and gene expression were obtained from the NCBI database. The differentially expressed genes (DEGs) and miRNAs (DEMs) were analyzed by GEO2R tools. The DEG-DEM interaction was built via miRNA-targeted genes by miRWalk. Several hub genes were selected via network topological analysis in Cytoscape. Results: A set of 276 genes were found to be significantly differentially expressed in the three datasets. Functional enrichment by the DAVID tool showed that these 276 DEGs were significantly enriched in the term “cancer”, with a statistic *p*-value of 1.9 × 10^−5^. The subdivision analysis of the specific cancer types indicated that “lung cancer” occupies the largest category with a *p*-value of 2 × 10^−3^. Furthermore, 75 miRNAs were shown to be differentially expressed in three representative datasets. A group of 13 DEGs was selected by analysis of the miRNA–gene interaction of these DEGs and DEMs. The investigation of these 13 genes by GEPIA tools showed that eight of them had consistent results with NSCLC samples in the TCGA database. In addition, we applied the KMplot to conduct the survival analysis of these eight genes and found that seven of them have a significant effect on the prognosis survival of patients. We believe that this study could provide effective research clues for the prevention and treatment of non-small cell lung cancer.

## 1. Introduction

Lung cancer is one of the most common diseases with the fastest increasing mortality rate and the greatest threat to human health. Lung cancer can be divided into small cell lung cancer (SCLC) and non-small cell lung cancer (NSCLC). In this study, we focus on NSCLC because this type is dominant in patients [1]. At present, lung adenocarcinoma and lung squamous cell carcinoma are the two major histopathological subtypes of NSCLC. Over 50% of NSCLC patients are diagnosed with stage IV lung cancer when the cancer is first detected [2]. The median overall survival of these patients ranges from 7–12 months depending on histology type [3]. It is necessary to understand the molecular mechanism of NSCLC progression, in order to predict and control this disease as early as possible.

Recently, much attention has been paid to miRNAs, which are small non-coding RNAs that play key roles in post-transcriptional regulation [4]. They are highly conserved between different species and are related to a series of basic cell processes. MiRNAs usually bind to 3′-UTR of the targeted mRNAs to inhibit the translation of targeted mRNAs. In fact, one miRNA can target a range of different genes with similar functions [5]. Many studies have reported the role of miRNAs in the regulation of NSCLC development, such as miR-224, miR-486 and miR-34a. It is demonstrated that miR-224 is up-regulated and promotes tumor progression and metastasis in NSCLC [6]. The miR-486 directly targets components related to insulin growth factor signaling and functions as a tumor suppressor in NSCLC [7]. The miR-34a is a direct transcriptional target of tumor-suppressor gene p53 and miR-34a expression is commonly regarded as contributing to tumorigenesis by attenuating p53-dependent apoptosis in NSCLC patients [8]. The results indicate that miRNA could be a suitable biomarker for diagnosis of this disease. However, most of these previous studies do not take the miRNA–gene interaction network into consideration. Systematic investigation of miRNA–gene interactions in NSCLC is still missing.

In this study, we identified differentially expressed genes and miRNAs in NSCLC samples. We establish a miRNA–gene interaction network, analyze their connection, and identify key biomarkers in this disease. We also perform survival analysis on the obtained biomarkers to verify our results. Based on these results, we can greatly promote the early prevention and treatment of non-small cell lung cancer.

## 2. Materials and Methods

### 2.1. Data Acquirement

The gene expression datasets were downloaded from the National Center for Biotechnology Information (NCBI) database. The keyword “non-small cell lung cancer” was searched in this database and the corresponding GEO datasets were recorded. To make the results more robust, we filtered the GEO datasets by the following criteria: (1) The dataset must include enough normal and tumor samples, i.e., total sample number ≥ 10; (2) The dataset must include enough gene/miRNA expression information. Based on these criteria, three GEO datasets (GSE18842, GSE101929, GSE29249) were retained for gene expression analysis, and another three datasets (GSE102286, GSE63805, GSE56036) were obtained for miRNA analysis.

### 2.2. Identification of DEG

In order to analyze the differences between normal samples and cancer samples, we used GEO2R tool [9] to analyze the three datasets (GSE18842, GSE101929, GSE29249) and obtained the differentially expressed genes (DEGs). The criteria for DEG were shown as follows: fold change ≥2 and *p*-value ≤ 0.05. We used the Perl program to summarize and analyze the differentially expressed genes of the three groups. Venn diagrams were drawn to better show our results.

### 2.3. Gene Ontology and Pathway Enrichment Analysis

Gene Ontology (GO) is a unique database which describes the characteristics and cell location of each gene [10]. KEGG is a database containing a large number of known metabolic pathways of genes [11]. DAVID is an online biological data tool, which can integrate the information of GO and pathways to analyze gene enrichment [12]. By DAVID enrichment analysis, we obtained the function group and keyword classification of our DEGs.

### 2.4. Genomic Specificity Analysis

ShinyGO is a graphical gene-set enrichment tool for model organisms [13]. We used ShinyGO to analyze the genomic information of these genes, including the number of exons, the length of UTR of the gene, the length of gene, etc. The significant differences in the genomic location were taken out for further analysis.

### 2.5. Regulatory Network Analysis

The STRING database is a commonly used protein–protein interaction database, which contains information on most species [14]. We used the STRING database to obtain the interaction information of DEGs. The interaction datasets are maintained for subsequent miRNA–gene interaction analysis.

### 2.6. Identification of Differentially Expressed miRNA

We obtained the datasets of miRNA expression in the NCBI database. Based on similar criteria in Section 2.1, three datasets (GSE102286, GSE63805 and GSE56036) were obtained in this study. Among them, GSE102286 contains 179 available samples, GSE63805 contains 66 available samples, and GSE56036 contains 12 available samples. We used the GEO2R tool to analyze the differentially expressed miRNA (DEMs) using these criteria: fold change ≥2 and *p*-value ≤ 0.05. We further used Venn diagrams to show the intersection of the three datasets.

### 2.7. Target Gene Analysis of miRNA

The miRWalk [15] is a commonly used software for miRNA target prediction and the tool was used to predict the interaction between miRNA and genes, using default parameters. We also used miRTarbase [16] to predict the target genes of miRNA. In addition, we applied miRPathDB [17] to analyze the metabolic pathway of target genes.

### 2.8. miRNA Gene Regulatory Network Analysis

The gene–gene interaction network obtained in Section 2.5 and the miRNA–gene interaction network obtained in Section 2.7 were integrated to construct a full miRNA–gene network. The software Cytoscape [18] was applied to visualize and analyze the network. Using the plugin Centiscape provided by Cytoscape, the node attributes in this network were obtained. Nodes with high network centrality were selected for subsequent analysis.

### 2.9. Verification Analysis by Independent Dataset

The nodes with high network centrality were selected for further verification. The TCGA datasets were used as third-party independent datasets to verify our results, which contain the next-generation sequencing data of all types of cancers. GEPIA server [19], which integrated the TCGA dataset analysis, was applied to analyze the gene expression information of our selected genes. In addition, the KMplot [20] was utilized to conduct the survival analysis of these genes, i.e., to check if these genes show a significant impact on the survival of NSCLC patients.

## 3. Results

### 3.1. Differentially Expressed Genes in NSCLC

**Figure 1:**
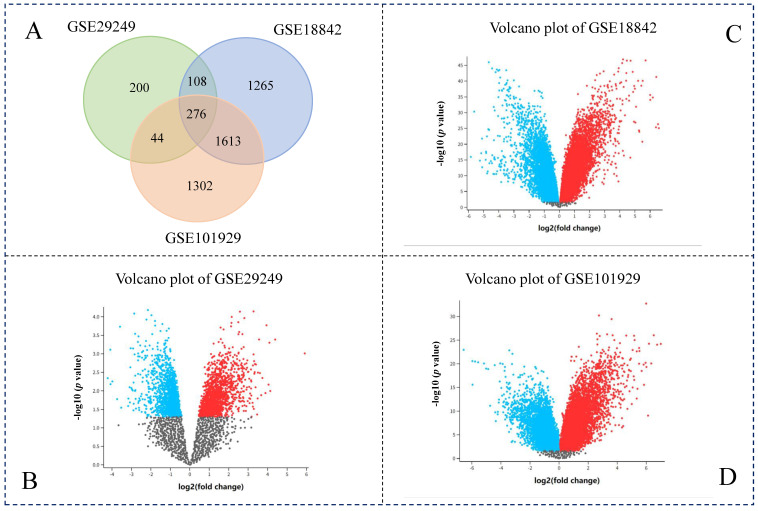
Analysis of differentially expressed genes in non-small cell lung cancer. (**A**) Venn diagram of differentially expressed miRNAs in three datasets; (**B**) Volcano plot of gene expression in GSE29249 dataset; (**C**) Volcano plot of gene expression in GSE18842 dataset; (**D**) Volcano plot of gene expression in GSE101929 dataset. The red dots indicated up-regulated, while the blue dots indicated the down-regulated.

We searched the GEO dataset of non-small cell lung cancer on the NCBI website and found three available datasets (GSE18842, GSE101929, GSE29249). The GEO2R tool was used to analyze these datasets to identify DEGs. We identified 628 DEGs from GSE29249, 3262 DEGs from GSE18842, and 3135 DEGs from GSE101929. Because GSE29249 has relatively few samples, the number of genes obtained is much smaller than the other two data sets (Figure 1). Through Venn diagram analysis, a set of 276 genes were found to overlap in these three datasets (Figure 1A).

### 3.2. Functional Enrichment Analysis of DEG

After obtaining DEGs, we performed the GO and KEGG enrichment to investigate the biological function of these 276 DEGs by using the DAVID database. The term “positive regulation of transcription from RNA polymerase II promoter” (GO:0045944) was significantly over-represented in our selected DEGs with a *p*-value = 6.2 × 10^−3^ in biological process, which indicated that these DEGs may function as transcription factors to regulate the downstream genes (Table S1). Furthermore, the term “Calcium Ion Binding” (GO:0005509) was significantly enriched in our DEGs with a *p*-value = 1.5 × 10^−5^ in molecular function analysis, which suggested that some of these DEGs could link to the tumor-suppressing pathway.

By GAD disease enrichment analysis of these DEGs, we found 86 (31.2%) genes could be significantly enriched in the keyword “Cancer”, with a significant *p*-value = 1.9 × 10^−5^ (Table 1). Detailed investigation of specific cancer types showed that the keyword “Lung cancer” occupied the largest proportion of different cancer types. Furthermore, 22 DEGs were significantly enriched in the keyword “Lung cancer”, with a *p*-value = 2.0 × 10^−3^. These results indicated that our identified DEGs are very likely related to NSCLC progression.

### 3.3. Genomic Specificity Analysis by ShinyGO

**Figure 2:**
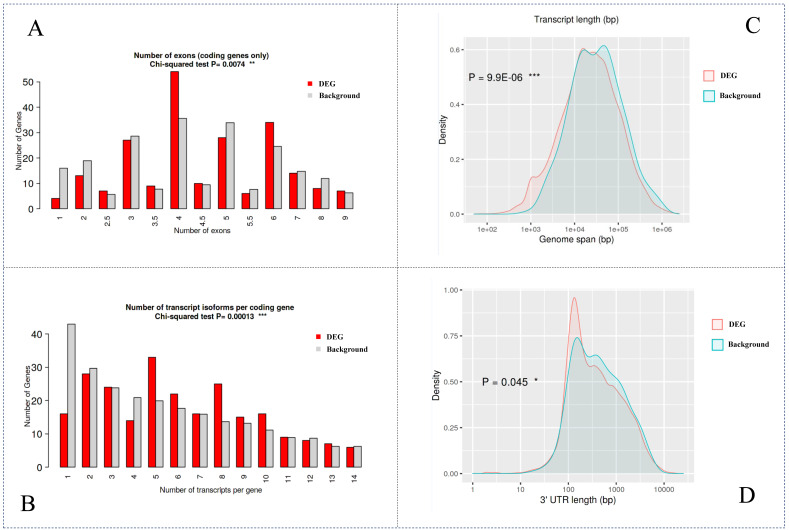
Genomic specificity analysis compared with the whole genome by ShinyGO. (**A**) Number of exons by chi-squared test; (**B**) Number of transcript isoforms per coding gene chi-squared test. (**C**) Transcript length compared with the whole genome; (**D**) 3′-UTR length compared with the whole genome. The symbol “*” indicated the significance level of the comparison.

We applied ShinyGO to analyze the genomic specificity of these DEGs when compared with the whole genome. We compared four aspects: number of exons, number of transcript isoforms per gene, genome span and 3′-UTR (untranslated region) length (Figure 2). Using the chi-squared test, the number of exons showed a significant *p*-value (0.0074) when comparing DEG with other genes in the genome. Besides, the number of transcript isoforms per gene was significantly different from the expected value with a *p*-value = 0.00013. These results indicated that our identified DEGs could have strong transcription characteristics with other genes, which might be involved in the cell proliferation of NSCLC patients. For the genome span analysis, we observed an extremely low *p*-value (9.9 × 10^−6^), while for 3′-UTR length comparison, we observed a relatively low *p*-value (0.045). Because 3′-UTR was the specific binding region of miRNA in the targeting gene, we suggest that the associated UTRs of these genes may alter the expression in the disease pathogenesis in NSCLC. 

### 3.4. PPI Visualization of DEGs

**Figure 3:**
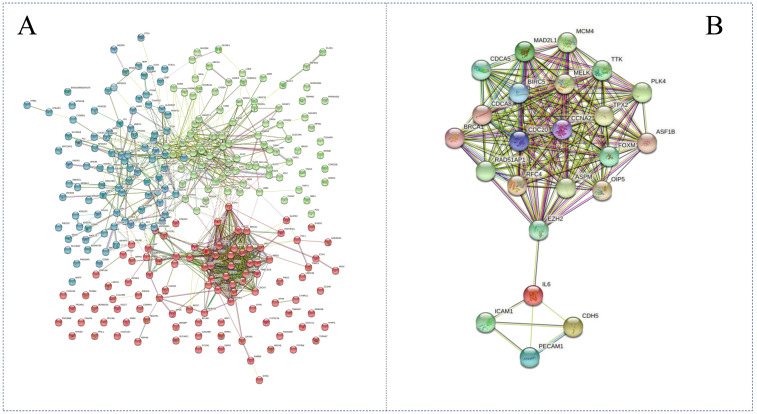
STRING network analysis of identified DEGs. (**A**) Full network of the DEGs. The different colors indicate the different groups in this figure. (**B**) Key part of the network. The colored nodes indicate the query proteins and the first shell of interactors; the white nodes indicate the second shell of interactors; the red edges indicate the experimentally determined protein–protein interactions; the green edges indicate the gene neighborhood of interaction; the black edges indicate the co-expression of interaction; the yellow edges indicate the text mining of interaction; the blue edges indicate the gene co-occurrence of interaction.

The PPI network was observed with 276 DEGs using the STRING database. A close relationship was visualized among these DEGs in this network. Using the Hidden Markov Model [21], these DEGs could be divided into three different groups (Figure 3A). It was shown that there was a highly associated relationship among BIRC5, MELK, CDC20, CCNA2 and EZH2 by node degree analysis. We suggested that there could be a clear positive correlation among these five gene clusters. To highlight the major regulation nodes in these DEGs, the genes were selected by STRING database and shown in Figure 3B. In addition, a strong association was indicated in other gene clusters (ICAM1, IL6, CDH5 and PECAM1). Two genes (EZH2 and IL6) were considered as intermediary hubs in these DEGs.

### 3.5. Identification of DEM

**Figure 4:**
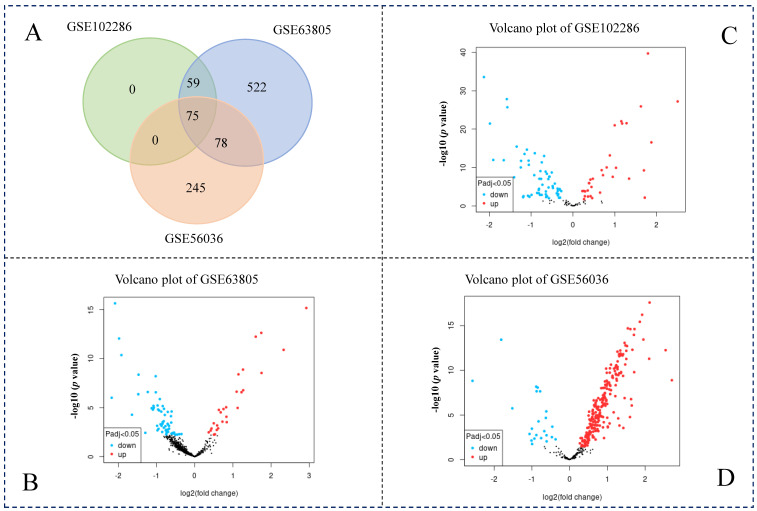
Analysis of differentially expressed miRNAs in non-small cell lung cancer. (**A**) Venn diagram of differentially expressed miRNAs in three datasets; (**B**) Volcano plot of gene expression in GSE63805 dataset; (**C**) Volcano plot of gene expression in GSE102286 dataset; (**D**) Volcano plot of gene expression in GSE56036 dataset. The red dots indicated up-regulated, while the blue dots indicated the down-regulated.

Similarly, three NSCLC datasets (GSE102286, GSE63805, GSE56036) with miRNA expression were downloaded from the NCBI database. Through GEO2R tools, we found that 134 miRNAs in GSE102286, 734 miRNAs in GSE63805, and 398 miRNAs in GSE56036 were significantly differentially expressed. Using Venn diagrams, 75 differentially expressed miRNAs (DEMs) were found overlapping in these three datasets (Figure 4). Although these datasets were sequenced in different times and different labs, most DEMs in GSE102286 can be found in the other two datasets (GSE63805, GSE56036).

### 3.6. DEG–DEM Interaction Prediction

miRWalk is known as an excellent tool for miRNA target prediction, thus this software was used to predict and analyze the regulatory interaction between 276 DEGs and 75 DEMs. As a result, 17 DEMs (miRNAs) were identified by miRWalk in the interaction. Characteristics of these miRNAs, including interacted DEGs (genes), protein ID and binding energy, are listed in Table 2. The identified 17 miRNAs were categorized into 13 different families based on the miRNA classification. Four miRNA families (miR-199, miR-361, miR-423, miR-574) were observed to have more than one member (hsa-miR-199a-5p, hsa-miR-199b-5p; hsa-miR-361-3p, hsa-miR-361-5p; hsa-miR-423-3p, hsa-miR-423-5p; hsa-miR-574-3p, hsa-miR-574-5p). The binding energy value, which suggests the Boltzmann-weighted probability to form a thermodynamically stable structure, ranged from −25.6 kcal/mol to −33.4 kcal/mol. Note that a low binding energy value is a critical parameter to discriminate miRNAs binding to targeted genes. Thus, these identified miRNAs were probably the true miRNA–gene interactions in NSCLC patients.

### 3.7. DEM Target Enrichment Analysis

The enrichment analysis of targeted genes of each DEM was conducted using the miRPathDB database. The targets of five miRNAs (hsa-miR-125a-5p, hsa-miR-331-3p, hsa-miR-199a-5p, hsa-miR-324-5p, hsa-miR-423-5p) were found to be enriched in the pathway of “Non-small cell lung cancer” (Table 3). A set of nine genes was identified as hsa-miR-125a-5p targets, with an extremely low *p*-value = 1.06 × 10^−5^. In addition, a total of 41 genes were identified as putative targets of hsa-miR-423-5p and these genes were significantly enriched in the pathway of “Non-small cell lung cancer” in WikiPathways Database [22], with a *p*-value = 0.023. These results suggest that the identified miRNAs are highly possible biomarkers in the progression of non-small cell lung cancer.

### 3.8. Network Analysis of the DEG-DEM Interaction

**Figure 5:**
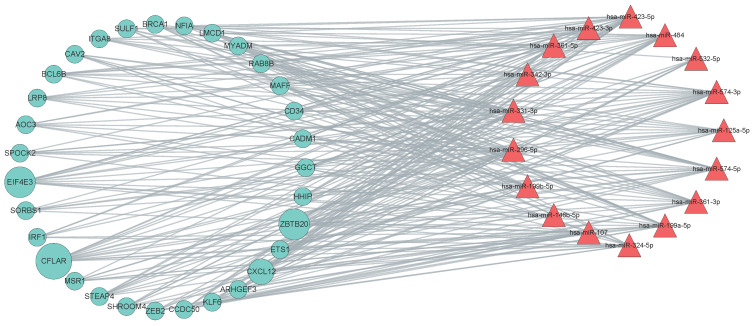
Network analysis of the interaction network between DEGs and DEMs. This figure only shows the interaction network of some important nodes. The red triangle represents miRNA; the green circle represents genes; the gray line represents the interaction. The size of circle (gene) reflects the degree centrality of the gene, i.e., a larger circle reflects more connections in this gene.

To identify key genes, the miRNA–gene interaction network was visualized by Cytoscape (Figure S1). The DEG and DEM in the network were categorized by their interactions. We analyzed the topology structure attribute, such as degree centrality and betweenness centrality of the interaction network using the plug-in toolkit Centiscape inside Cytoscape. Some nodes with higher centrality were selected and were used to re-draw the core network (Figure 5). The results show that genes and miRNA can form a relatively independent module, and these modules as a whole may play an indelible role in the occurrence and development of NSCLC. In addition, based on the degree centrality of the network and miRNA–gene interaction information of miRTarBase database, we screened out 13 key genes (AKAP13, ANAX11, CAD, ETS1, GGCT, HHIP, KCNK3, KLF2, OLR1, PPIL1, SBK1, TWIST1, ZBTB20) and nine key miRNAs (hsa-miR-423-5p, hsa-miR-484, hsa-miR-331-3p, hsa-miR-125a-5p, hsa-miR-574-5p, hsa-miR-361-5p, hsa-miR-361-3p, hsa-miR-199a-5p, hsa-miR-324-5p) (Table S1). These genes and miRNAs could be the hub molecules in the DEG–DEM interaction network of NSCLC, which need further verification in the next steps.

### 3.9. Validation of Hub Genes Using GEPIA

**Figure 6:**
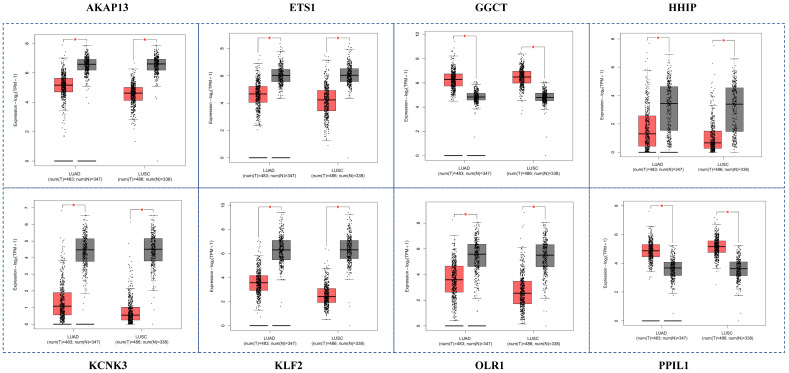
The validation of hub genes by the GEPIA. Here, we only show eight genes with a *p*-value ≤ 0.05. In this figure, NSCLC is divided into two types: lung adenocarcinoma (LUAD) and lung squamous cell carcinoma (LUSC). The symbol “*” indicated the significance level of the comparison.

In the above paragraph, we screened out 13 key genes in Cytoscape. GEPIA website collected a large number of cancer samples from TCGA database, which could be used to verify the reliability of selected 13 genes. Results indicated that eight genes (AKAP13, ETS1, GGCT, HHIP, KCNK3, KLF2, OLR1, PPIL1) showed significant differential expression between normal samples and NSCLC samples in TCGA database with a *p*-value ≤ 0.05 (Figure 6). Furthermore, we drew a scatter plot of the expression levels of these 13 genes for all cancer types. Results showed that the expression levels of OLR1 and HHIP genes in NSCLC were much higher than in other cancer types (Figure S2), indicating that these two genes could serve as specific biomarkers in NSCLC progression.

### 3.10. Survival Analysis of Hub Genes Using KMplot

**Figure 7:**
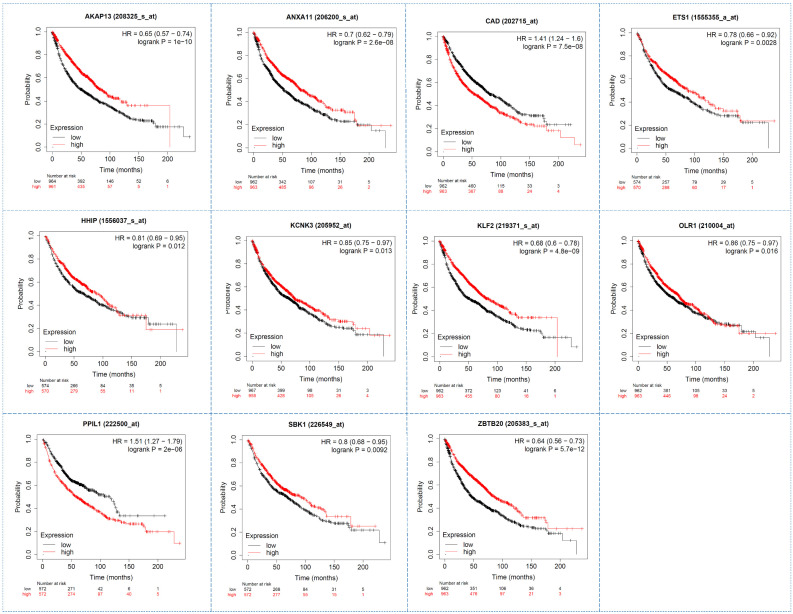
Survival analysis of hub genes by the KMplot tool. Here, we only show 11 genes with a significant *p*-value ≤0.05.

Survival analysis is a basic medical research method, which can be used to assess clinical outcomes for treatment efficiency and disease progression. The KMplot is a useful tool to conduct the survival analysis for the selected 13 DEGs. We found that 11 genes (AKAP13, ANAX11, CAD, ETS1, HHIP, KCNK3, KLF2, OLR1, PPIL1, SBK1, ZBTB20) showed a significant impact on the survival of NSCLC patients with a *p*-value ≤ 0.05 (Figure 7). Among them, seven genes (AKAP13, ETS1, HHIP, KCNK3, KLF2, OLR1, PPIL1) satisfy the above-mentioned criteria of both GEPIA and KMplot. We believe that these seven genes are the critical molecules in the development of NSCLC and can be used as reliable biomarkers in the early prevention of NSCLC patients.

## 4. Discussion

Non-small cell lung cancer (NSCLC) is one of the most common types of lung cancer, which may resist most radiotherapy and chemotherapy treatments. Currently, biomarker-targeting therapy is still the first-line therapy for advanced NSCLC patients. It is highly important to explore the possible mechanisms of NSCLC carcinogenesis and discover reliable biomarkers for early diagnosis. These biomarkers could serve as novel molecular targets for predicting the prognosis of NSCLC patients.

Recently, much attention has been paid to miRNAs, which play a critical role in the development of various types of cancers. It has been reported that abnormally expressed miRNAs are found in NSCLC proliferation and metastasis, such as let-7c and miR-218. The miRNA let-7c, a member of the let-7 family, prevents migration and invasion of NSCLC cells by degrading oncogene ITGB3 and could be used as a tumor suppressor in this type of cancer [23]. Furthermore, scientists have reported that overexpression of miR-218 in NSCLC cells inhibits cell invasion and proliferation by targeting the IL-6 receptor [24]. These results indicate that miRNA dysregulation could be used as an early diagnosis signal for the detection of NSCLC. However, searching for more effective miRNA-targeting genes might assist in better understanding the pathogenesis of NSCLC.

In this study, we obtained three miRNA-oriented datasets (GSE102286, GSE63805, GSE56036) and three gene-oriented datasets (GSE18842, GSE101929, GSE29249) from GEO database. The miRNAs and genes were screened between NSCLC and adjacent normal tissues in these six datasets by bioinformatics analysis. A set of 75 DEMs and 276 DEGs were identified in the corresponding datasets, respectively. 

To establish the connection between the 75 DEMs and 276 DEGs, we built a DEM–DEG interaction information by miRWalk. Fortunately, through analysis of miRNA–gene interactions, we found nine miRNAs could build bridges with 13 genes. By miRNA target enrichment analysis by miRPathDB, five miRNAs (hsa-miR-125a-5p, hsa-miR-331-3p, hsa-miR-199a-5p, hsa-miR-324-5p, hsa-miR-423-5p) were found to be enriched in the pathway of “Non-small cell lung cancer”. Through gene expression analysis by GEPIA and survival analysis by KMplot, seven genes (AKAP13, ETS1, HHIP, KCNK3, KLF2, OLR1, PPIL1) were further screened and retained. These genes and miRNAs could be highly probably used as novel biomarkers for early diagnosis in NSCLC patients. The following are some examples for detailed description.

Hsa-miR-125a-5p has been previously reported to be downregulated in various lung cancer types and has been validated to prevent cancer cell progression [25]. Scientists have demonstrated that abnormal expression of hsa-miR-125a-5p is involved in lung cancer metastasis by targeting PTPRU and this miRNA is a predictor for patients with advanced NSCLC. A similar phenomenon has also been discovered towards hsa-miR-324-5p. Scientists have found that hsa-miR-324-5p could be used as a unique miRNA signature for NSCLC. Overexpression of hsa-miR-324-5p could activate FBXO11 signaling and potentiate resistance to cisplatin in NSCLC cells [26].

The AKAP13 gene, located in human chromosome 15, is reported to be involved in the pathogenesis of various cancers, including NSCLC. A previous study reported that AKAP13 protein contributes to loss of E-cadherin and the bronchial epithelial barrier in NSCLC cells [27]. KCNK3, also known as TASK-1, is expressed in NSCLC cell lines at variable levels. Inhibition of KCNK3 leads to significant depolarization in these cells [28]. KLF2, a member of the KLF family, also known as lung Krüppel-like factor, was reported to be highly expressed in normal lung tissue of embryo and to be essential for later development of embryonic lung [29]. KLF2 can modulate the expression of many downstream genes by binding to the GC-enriched regions of gene promoters.

Based on the above analysis, we have high confidence that our identified genes and miRNAs can be used as novel biomarkers for diagnosis and prognosis of NSCLC. Our study has important significance for better understanding the development and prognosis of this disease.

## 5. Conclusions

We applied a series of bioinformatics tools to analyze the gene and miRNA expression datasets of NSCLC samples. Through a series of analyses of miRNA–gene interactions, we screened seven genes and five miRNAs which could be used as novel biomarkers for diagnosis of this disease. These biomarkers provide useful clues for future research on the development and prognosis of NSCLC.

## Figures and Tables

**Table 1:** Functional enrichment analysis of DEGs by DAVID.

Category	Term	Gene Number	Count	Percent (%)	*p*-Value
GAD_DISEASE_CLASS	CANCER	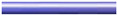	86	31.2	1.90 × 10^−^^5^
	---Lung Cancer		22	8	2.00 × 10^−^^3^
	---Cervical cancer		7	2.5	4.50 × 10^−^^3^
	---Breast Cancer		21	7.6	4.90 × 10^−^^3^
	---Prostate cancer		18	6.5	9.50 × 10^−^^3^
GAD_DISEASE_CLASS	RENAL	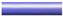	46	16.7	7.00 × 10^−^^5^
GAD_DISEASE_CLASS	UNKNOWN	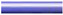	47	17	6.80 × 10^−^^4^
GAD_DISEASE_CLASS	CARDIOVASCULAR	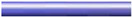	100	36.2	7.40 × 10^−^^4^
GAD_DISEASE_CLASS	REPRODUCTION	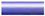	31	11.2	1.70 × 10^−^^3^
GAD_DISEASE_CLASS	IMMUNE	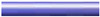	72	26.1	3.50 × 10^−^^3^
GAD_DISEASE_CLASS	DEVELOPMENTAL	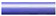	41	14.9	5.50 × 10^−^^3^
GAD_DISEASE_CLASS	OTHER	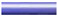	42	15.2	8.90 × 10^−^^3^

**Table 2:** DEG–DEM interaction prediction. This table only shows one interaction of one DEM, i.e., part of the results. The full table is shown in the Supplementary Materials (Table S2).

No.	DEM (miRNA)	DEG (Gene)	Protein ID	Binding Energy
				(kcal/mol)
1	hsa-miR-484	KDELR2	NM_006854	−33.4
2	hsa-miR-574-5p	STEAP4	NM_001205316	−33
3	hsa-miR-331-3p	LMCD1	NM_001278233	−32.6
4	hsa-miR-324-5p	CFLAR	NM_001308043	−31.8
5	hsa-miR-423-3p	LRRC32	NM_001128922	−31.7
6	hsa-miR-423-5p	GPI	NM_001289789	−31.2
7	hsa-miR-125a-5p	COLEC12	NM_130386	−31
8	hsa-miR-296-5p	GRK5	NM_005308	−30.6
9	hsa-miR-361-5p	ARHGEF3	NM_001289698	−30.5
10	hsa-miR-574-3p	GLIPR2	NM_001287014	−29.5
11	hsa-miR-107	CCDC50	NM_174908	−27.7
12	hsa-miR-342-3p	SLC11A1	NM_000578	−27.4
13	hsa-miR-361-3p	CXCL12	NM_000609	−27.1
14	hsa-miR-199b-5p	MAFF	NM_012323	−26.4
15	hsa-miR-199a-5p	MAFF	NM_012323	−26.3
16	hsa-miR-146b-5p	TIMELESS	NM_001330295	−25.7
17	hsa-miR-532-5p	CSRNP1	NM_001320560	−25.6

**Table 3:** Enrichment analysis of DEM-targeted genes by miRPathDB.

miRNA	Database	Pathway	Targeted Gene Hits	Expected Hits	*p*-Value
hsa-miR-125a-5p	WikiPathways	Non-small cell lung cancer	9	0.997	1.06 × 10^−5^
hsa-miR-331-3p	WikiPathways	Non-small cell lung cancer	3	0.156	0.002
hsa-miR-199a-5p	WikiPathways	Non-small cell lung cancer	4	0.705	0.014
hsa-miR-423-5p	WikiPathways	Non-small cell lung cancer	41	28.09	0.023
hsa-miR-324-5p	KEGG	Non-small cell lung cancer	23	13.86	0.046

## Data Availability

All data are available from the GEO repository (https://www.ncbi.nlm.nih.gov/gds) (accessed on 1 September 2021).

## Supplementary Materials

The following supporting information can be downloaded at: https://www.mdpi.com/article/10.3390/genes13081480/s1, Figure S1: Full view of the interaction network between DEGs and DEMs; Figure S2: scatter plot of expression levels of OLR1 and HHIP; Table S1: Gene ontology enrichment by DAVID; Table S2: full table of DEG–DEM interaction predictions.

## References

[1] Wu S.Y., Pan Y., Mao Y.Y., Chen Y., He Y.Y. (2021). Current progress and mechanisms of bone metastasis in lung cancer: A narrative review. Transl. Lung Cancer R.

[2] Hanna N.H., Robinson A.G., Temin S., Baker S., Brahmer J.R., Ellis P.M., Gaspar L.E., Haddad R.Y., Hesketh P.J., Jain D. (2021). Therapy for stage IV non–small-cell lung cancer with driver alterations: ASCO and OH (CCO) joint guideline update. J. Clin. Oncol..

[3] Chhatre S., Vachani A., Allison R.R., Jayadevappa R. (2021). Survival Outcomes with Photodynamic Therapy, Chemotherapy and Radiation in Patients with Stage III or Stage IV Non-Small Cell Lung Cancer. Cancers.

[4] Yang Z., Wang M., Zeng X., Wan A.T.-Y., Tsui S.K.-W. (2020). In silico analysis of proteins and microRNAs related to human African trypanosomiasis in tsetse fly. Comput. Biol. Chem..

[5] Yang Z., Wan A.T.Y., Liu X.Y., Xiong Q., Liu Z.G., Tsui S.K.W. (2019). Identification, functional annotation and stability analysis of miRNA in Dermatophagoides pteronyssinus. Allergy.

[6] Li S., Zhang J.G., Zhao Y.W., Wang F.L., Chen Y., Fei X.B. (2018). miR-224 enhances invasion and metastasis by targeting HOXD10 in non-small cell lung cancer cells. Oncol. Lett..

[7] Gao Z.J., Yuan W.D., Yuan J.Q., Yuan K., Wang Y. (2018). miR-486-5p functions as an oncogene by targeting PTEN in non-small cell lung cancer. Pathol. Res. Pract..

[8] Xiong R., Sun X.X., Wu H.R., Xu G.W., Wang G.X., Sun X.H., Xu M.Q., Xie M.R. (2020). Mechanism research of miR-34a regulates Axl in non-small-cell lung cancer with gefitinib-acquired resistance. Thorac. Cancer.

[9] Barrett T., Wilhite S.E., Ledoux P., Evangelista C., Kim I.F., Tomashevsky M., Marshall K.A., Phillippy K.H., Sherman P.M., Holko M. (2013). NCBI GEO: Archive for functional genomics data sets-update. Nucleic Acids Res..

[10] Consortium G.O. (2019). The gene ontology resource: 20 years and still GOing strong. Nucleic Acids Res..

[11] Kanehisa M., Sato Y., Kawashima M. (2022). KEGG mapping tools for uncovering hidden features in biological data. Protein Sci..

[12] Sherman B.T., Hao M., Qiu J., Jiao X.L., Baseler M.W., Lane H.C., Imamichi T., Chang W.Z. (2022). DAVID: A web server for functional enrichment analysis and functional annotation of gene lists (2021 update). Nucleic Acids Res..

[13] Ge S.X., Jung D.M., Yao R.A. (2020). ShinyGO: A graphical gene-set enrichment tool for animals and plants. Bioinformatics.

[14] Szklarczyk D., Gable A.L., Nastou K.C., Lyon D., Kirsch R., Pyysalo S., Doncheva N.T., Legeay M., Fang T., Bork P. (2021). The STRING database in 2021: Customizable protein-protein networks, and functional characterization of user-uploaded gene/measurement sets (vol 49, pg D605, 2021). Nucleic Acids Res..

[15] Sticht C., De La Torre C., Parveen A., Gretz N. (2018). miRWalk: An online resource for prediction of microRNA binding sites. PLoS ONE.

[16] Huang H.Y., Lin Y.C.D., Cui S.D., Huang Y.X., Tang Y., Xu J.T., Bao J.Y., Li Y.L., Wen J., Zuo H.L. (2022). miRTarBase update 2022: An informative resource for experimentally validated miRNA-target interactions. Nucleic Acids Res..

[17] Kehl T., Kern F., Backes C., Fehlmann T., Stockel D., Meese E., Lenhof H.P., Keller A. (2020). miRPathDB 2.0: A novel release of the miRNA Pathway Dictionary Database. Nucleic Acids Res..

[18] Doncheva N.T., Morris J.H., Gorodkin J., Jensen L.J. (2019). Cytoscape StringApp: Network Analysis and Visualization of Proteomics Data. J. Proteome Res..

[19] Li C.W., Tang Z.F., Zhang W.J., Ye Z.C., Liu F.L. (2021). GEPIA2021: Integrating multiple deconvolution-based analysis into GEPIA. Nucleic Acids Res..

[20] Lanczky A., Gyorffy B. (2021). Web-Based Survival Analysis Tool Tailored for Medical Research (KMplot): Development and Implementation. J. Med. Internet. Res..

[21] Lei X., Wang F., Wu F.-X., Zhang A., Pedrycz W. (2016). Protein complex identification through Markov clustering with firefly algorithm on dynamic protein–protein interaction networks. Inf. Sci..

[22] Martens M., Ammar A., Riutta A., Waagmeester A., Slenter D.N., Hanspers K., Miller R.A., Digles D., Lopes E.N., Ehrhart F. (2021). WikiPathways: Connecting communities. Nucleic Acids Res..

[23] Zhao B., Han H., Chen J., Zhang Z., Li S., Fang F., Zheng Q., Ma Y., Zhang J., Wu N. (2014). MicroRNA let-7c inhibits migration and invasion of human non-small cell lung cancer by targeting ITGB3 and MAP4K3. Cancer Lett..

[24] Yang Y., Ding L., Hu Q., Xia J., Sun J., Wang X., Xiong H., Gurbani D., Li L., Liu Y. (2017). MicroRNA-218 functions as a tumor suppressor in lung cancer by targeting IL-6/STAT3 and negatively correlates with poor prognosis. Mol. Cancer.

[25] Huang H., Huang J.Y., Yao J., Li N., Yang Z.Z. (2020). miR-125a regulates HAS1 and inhibits the proliferation, invasion and metastasis by targeting STAT3 in non-small cell lung cancer cells. J. Cell Biochem..

[26] Ba Z., Zhou Y., Yang Z., Xu J., Zhang X. (2019). miR-324-5p upregulation potentiates resistance to cisplatin by targeting FBXO11 signalling in non-small cell lung cancer cells. J. Biochem..

[27] Wang H.L., Li K.Z., Li J.L., Hu B.L. (2020). Prognostic value of AKAP13 methylation and expression in lung squamous cell carcinoma. Biomark. Med..

[28] Leithner K., Hirschmugl B., Li Y.J., Tang B., Papp R., Nagaraj C., Stacher E., Stiegler P., Lindenmann J., Olschewski A. (2016). TASK-1 Regulates Apoptosis and Proliferation in a Subset of Non-Small Cell Lung Cancers. PLoS ONE.

[29] Jiang W.B., Xu X.Q., Deng S.L., Luo J., Xu H., Wang C., Sun T.T., Lei G.Q., Zhang F.L., Yang C. (2017). Methylation of kruppel-like factor 2 (KLF2) associates with its expression and non-small cell lung cancer progression. Am. J. Trans. Res..

