# Supplementary figures and images for: Gene–microRNA Network Analysis Identified Seven Hub Genes in Association with Progression and Prognosis in Non-Small Cell Lung Cancer
Source: Genes (Basel). 2022 Aug 19;13(8):1480. doi: 10.3390/genes13081480 (PMC9407881; doi:10.3390/genes13081480)

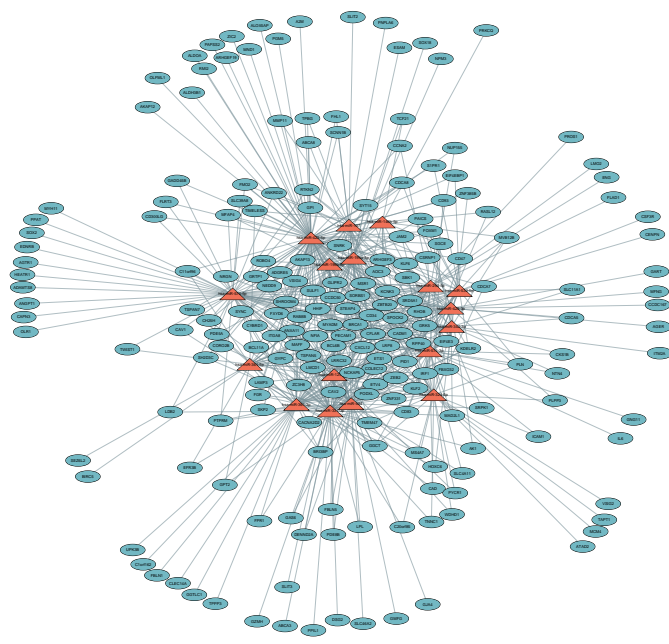

Supplement: Supplementary file 1 [file genes-13-01480-s001.zip › Figure S1 - Full view of the interaction network between DEGs and DEMs.pdf]

Transcripts Per Million (TPM)

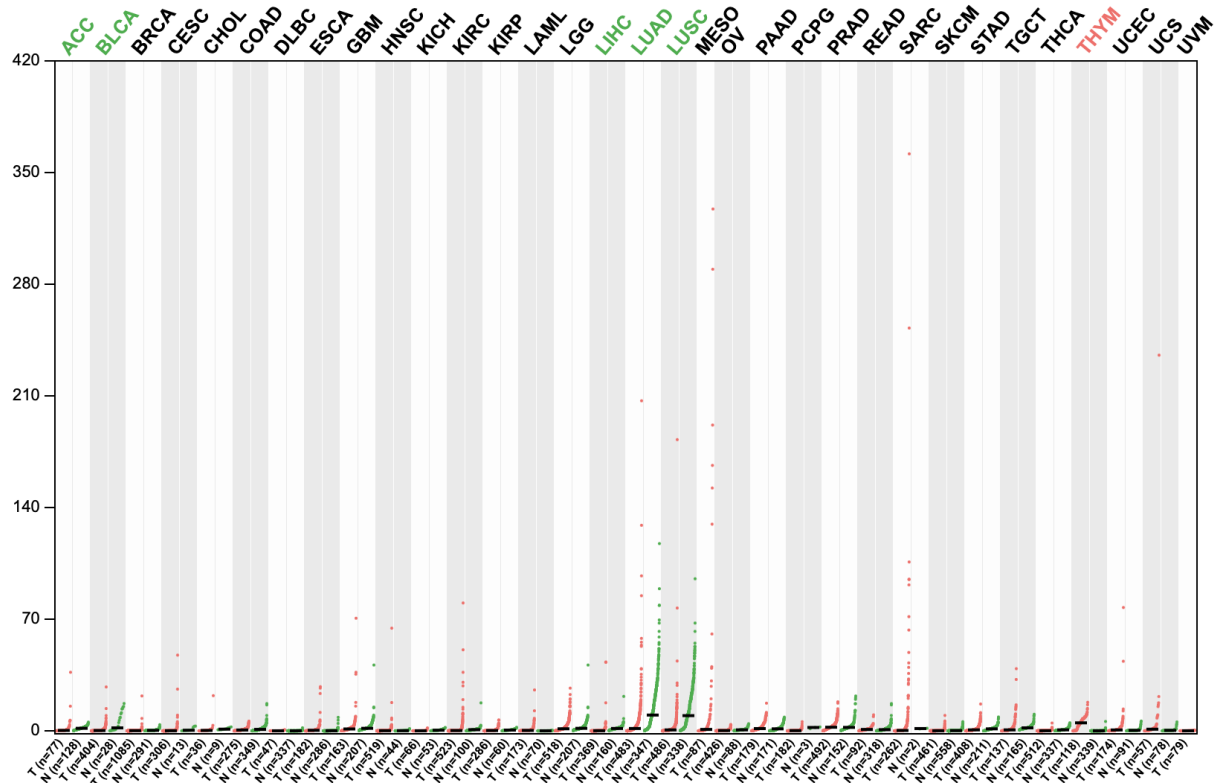

Transcripts Per Million (TPM)

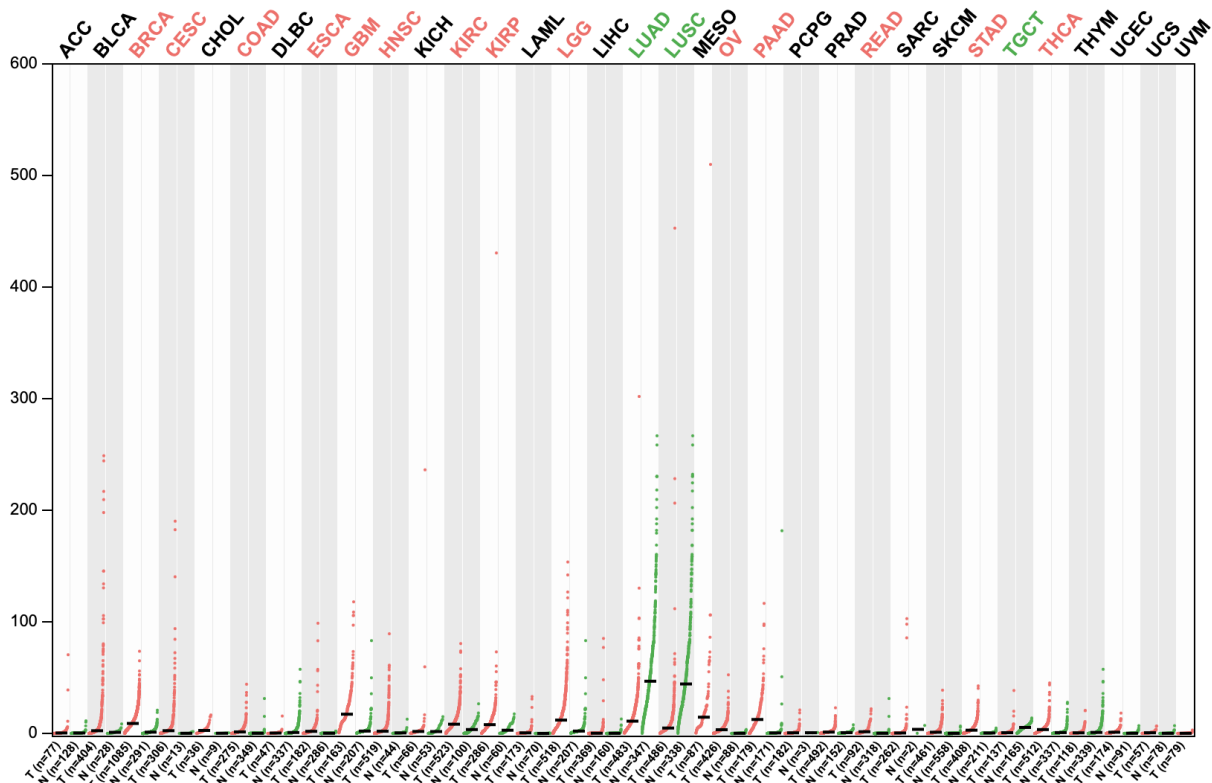

Supplement: Supplementary file 1 [file genes-13-01480-s001.zip › Figure S2 - scatter plot of expression levels of OLR1 and HHIP .pdf]
